# Gastrointestinal Stromal Tumors in the Stomach With Tumor Growth and Hemorrhage During Conservative Management: A Report of Two Cases

**DOI:** 10.7759/cureus.82046

**Published:** 2025-04-10

**Authors:** Masaya Iwamuro, Satoru Kikuchi, Shinji Kuroda, Takehiro Tanaka, Motoyuki Otsuka

**Affiliations:** 1 Department of Gastroenterology and Hepatology, Okayama University Graduate School of Medicine, Dentistry, and Pharmaceutical Sciences, Okayama, JPN; 2 Department of Gastroenterological Surgery, Okayama University Graduate School of Medicine, Dentistry, and Pharmaceutical Sciences, Okayama, JPN; 3 Department of Pathology, Okayama University Hospital, Okayama, JPN

**Keywords:** conservative management, gastric subepithelial lesion, gastrointestinal bleeding, gastrointestinal stromal tumor, tumor growth

## Abstract

Gastrointestinal stromal tumors (GISTs) are often detected incidentally during esophagogastroduodenoscopy. Although surgical resection is the standard treatment for GISTs, patients with significant comorbidities may not be eligible for surgery and are managed conservatively. Herein, we report two cases of gastric GISTs that were initially observed during the management of other comorbidities but subsequently became enlarged, resulting in gastrointestinal bleeding. These cases highlight the potential risks of tumor progression and bleeding in patients undergoing conservative management.

## Introduction

Gastric subepithelial lesions (SELs) are frequently detected during endoscopic examinations, and most patients are asymptomatic. Among these, gastrointestinal stromal tumors (GISTs) are of particular clinical significance due to their potential for malignant transformation and growth [1]. Although surgical resection is the standard treatment, GISTs can occasionally be managed conservatively in patients with significant comorbidities that preclude surgical intervention [2-4]. GISTs have an inherent potential for growth [5], and their natural course under observation remains poorly documented, particularly in cases where surgery is not feasible owing to patient-related factors. In such instances, careful long-term follow-up is crucial, as tumor enlargement may lead to complications such as hemorrhage.

Here, we present two cases of gastric GISTs that were initially observed during the management of other comorbidities, but later exhibited progressive growth and resulted in gastrointestinal bleeding. These cases highlight the challenges of conservative management of high-risk patients and underscore the need for close surveillance to prevent potential complications.

## Case presentation

Case 1

An 82-year-old Japanese woman underwent esophagogastroduodenoscopy at her primary care clinic as part of a routine health checkup; this revealed a SEL in the stomach. One year later, a follow-up endoscopy revealed tumor enlargement, and the patient was referred to our hospital for further evaluation. She had a history of atrial fibrillation and was on warfarin therapy. Additionally, the patient was receiving oral medication for hypertension and hyperlipidemia, and had a history of femoral neck fracture and total hysterectomy due to a uterine abscess.

**Figure 1:**
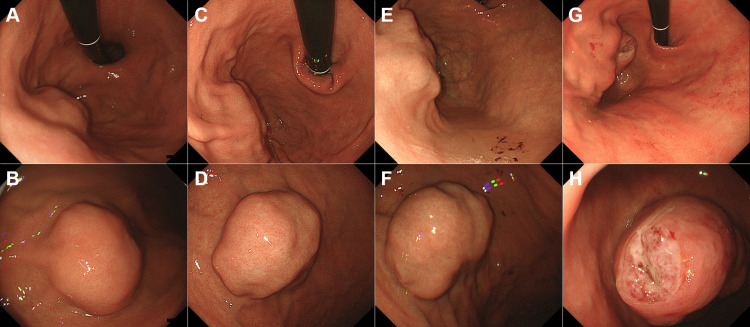
Initial and follow-up endoscopic findings of case 1. Initial esophagogastroduodenoscopy showing a 25 mm elevated lesion on the greater curvature of the upper gastric body (A and B). Follow-up endoscopy images taken one (C and D) and two (E and F) years showing gradual tumor enlargement. At three years of follow-up, endoscopy revealed significant tumor growth with multiple ulcerations and an irregular surface (G and H).

**Figure 2:**
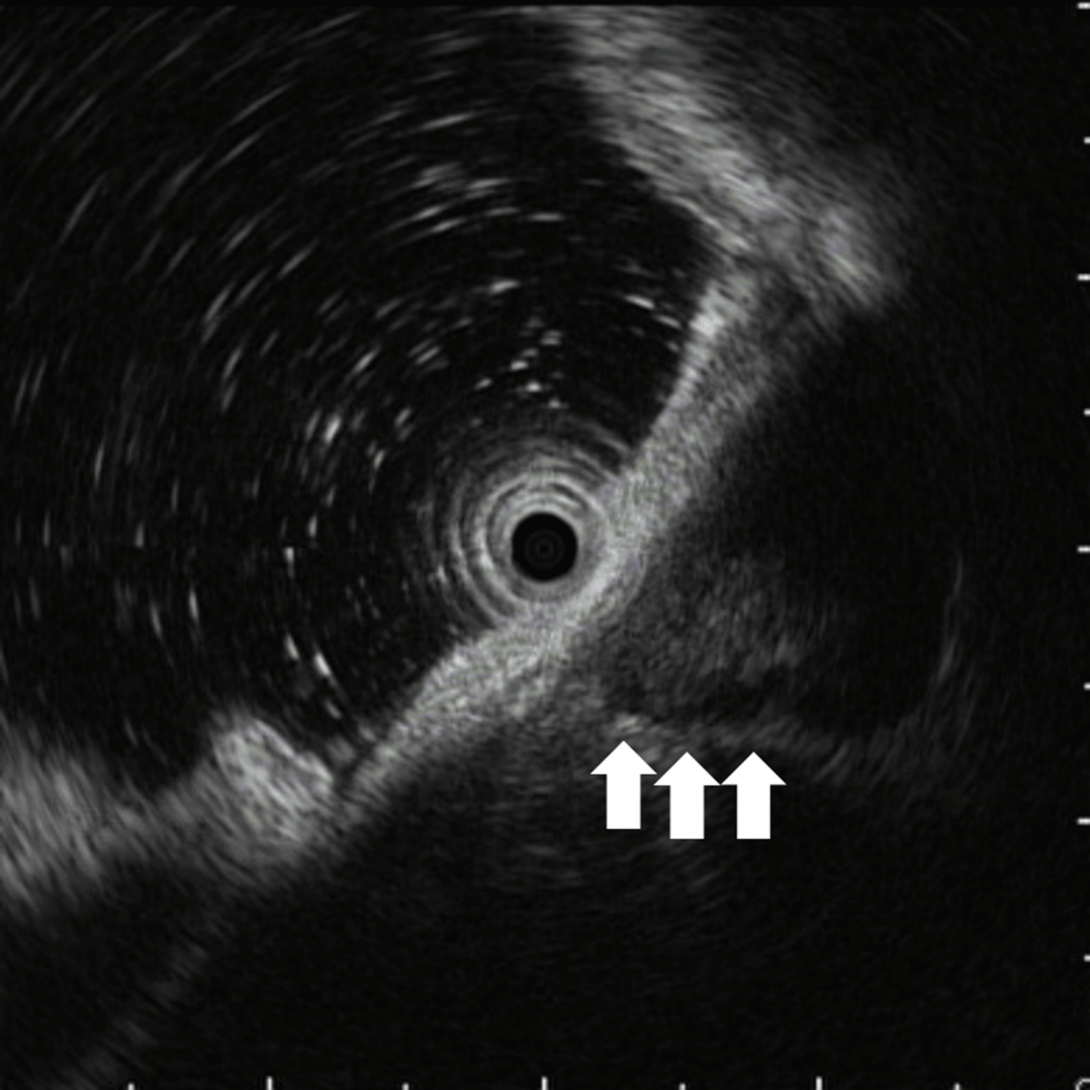
Endoscopic ultrasonography of case 1. Endoscopic ultrasonography showing a heterogeneous internal echo pattern and an irregular tumor margin (arrows), consistent with a gastrointestinal stromal tumor. The tumor originated from the fourth layer of the gastric wall.

**Figure 3:**
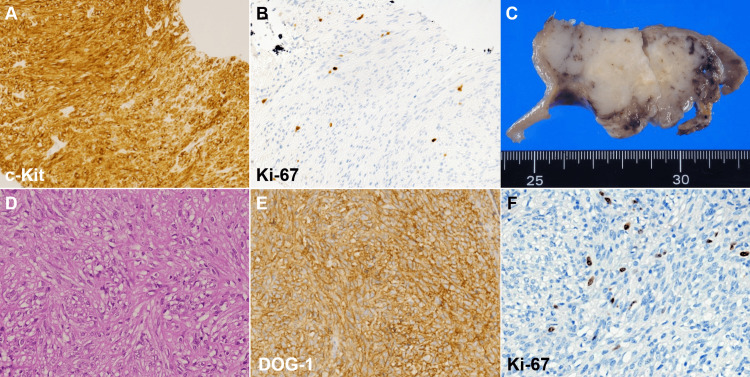
Histopathological and surgical findings of case 1. Histopathological examination of the endoscopic ultrasound-guided fine-needle aspiration specimen revealed spindle-shaped cells positive for c-Kit, confirming a diagnosis of gastrointestinal stromal tumor (GIST) (A). The Ki-67 labeling index was 1.9% (B). A cut section of the laparoscopically resected tumor showed a 65 mm mass with surrounding ulceration and necrosis (C). Pathological examination revealed nodular proliferation of spindle-shaped cells (D). Immunohistochemical staining demonstrated positive DOG-1 expression (E). The Ki-67 labeling index was 9.7% (F).

Esophagogastroduodenoscopy revealed a 25 mm elevated lesion on the greater curvature of the upper gastric body, with no apparent surface mucosal irregularities (Figure 1A, 1B). The lesion had a steep elevation with a bridging fold, suggestive of a SEL, which was firm upon compression. Endoscopic ultrasonography demonstrated that the tumor was continuous from the fourth layer of the gastric wall, exhibited an irregular border at the tumor margin (Figure 2, arrows), and exhibited a heterogeneous internal echo pattern. Endoscopic ultrasound-guided fine-needle aspiration confirmed a diagnosis of GIST (Figure 3A). Histopathologically, no mitotic figures were observed, and the Ki-67 labeling index was 1.9% (Figure 3B). The patient was scheduled to undergo GIST resection via laparoscopic and endoscopic cooperative surgery; however, she developed cerebral infarction immediately before the operation. Consequently, GIST resection was not performed, leading to conservative management. The patient underwent an endoscopic examination every year, and although the GIST gradually increased in size over time (Figure 1C-1F), the patient remained asymptomatic. Therefore, follow-up was continued.

Three years after the GIST diagnosis, she was referred to our hospital because of anemia and melena. Esophagogastroduodenoscopy revealed significant tumor growth and multiple ulcerations (Figure 1G, 1H). A laparoscopic partial gastrectomy was performed, and the tumor measured 65 mm in diameter (Figure 3C). Pathologically, the nodular proliferation of spindle-shaped cells was observed (Figure 3D). These cells were positive for c-Kit and DOG-1 (Figure 3E), leading to a final diagnosis of GIST. Mitotic count was 16 per 50 high-power fields, and the Ki-67 labeling index was 9.7% (Figure 3F).

Case 2

An 81-year-old Japanese man underwent esophagogastroduodenoscopy for heartburn evaluation, which revealed reflux esophagitis and a 15 mm SEL in the stomach. The patient had hypertension, hyperlipidemia, cataracts, and a history of ureteral stone formation. He had been taking cilostazol, limaprost alfadex, allopurinol, naftopidil, acetylleucine, losartan, hydrochlorothiazide, pravastatin, and pregabalin for cerebral infarction, internal carotid artery stenosis, hyperuricemia, benign prostatic hyperplasia, vertigo, hypertension, dyslipidemia, and neuropathic pain, respectively. Follow-up endoscopy was performed every six months for gastric SEL, and the lesion enlarged to 30 mm two years later, prompting a referral to our hospital for further evaluation.

**Figure 4:**
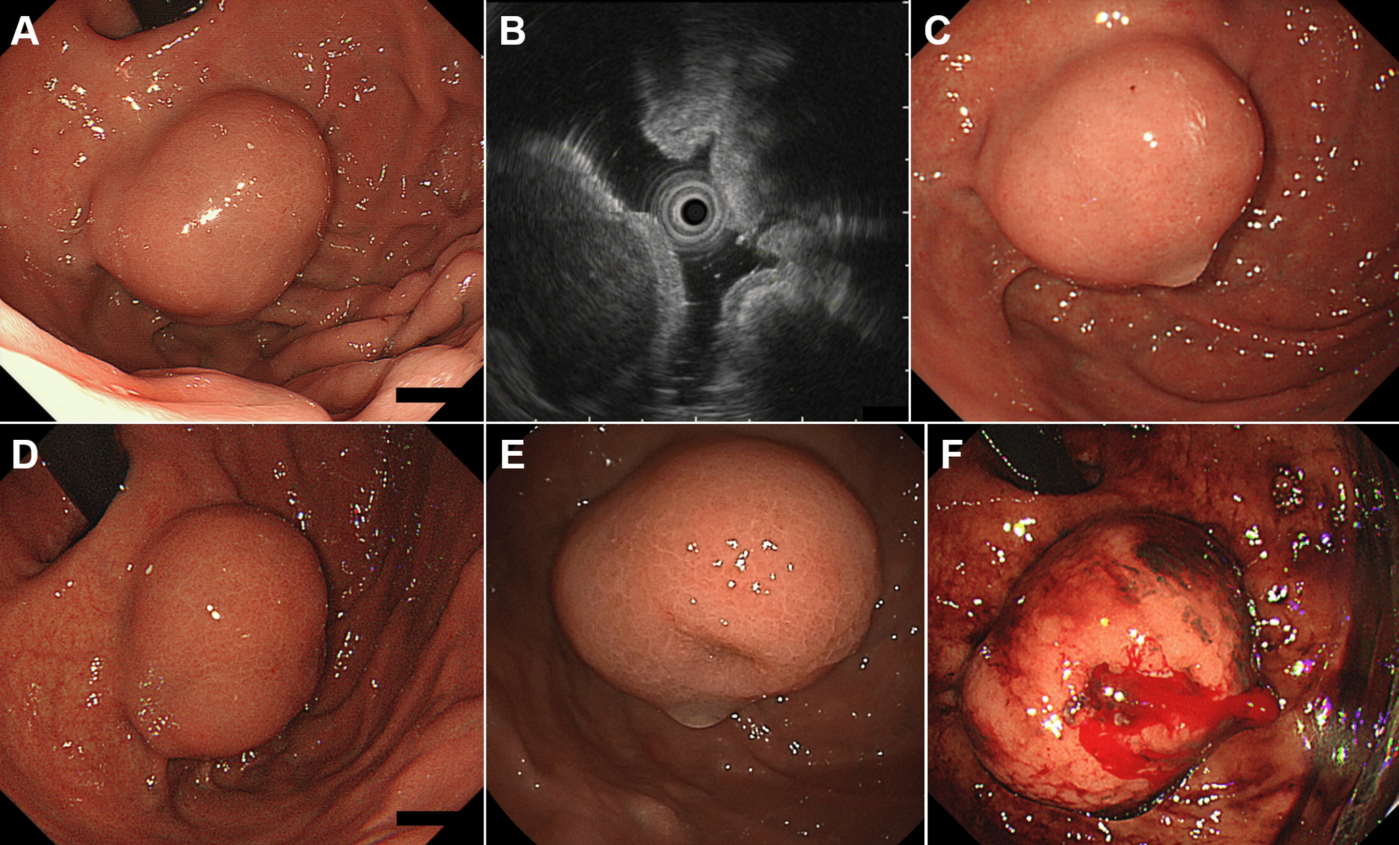
Case 2: initial and follow-up endoscopic findings of a gastric subepithelial lesion (SEL). Initial esophagogastroduodenoscopy (EGD) at the first visit showing a 15 mm SEL in the gastric fundus, with firm elasticity at palpation (A). Endoscopic ultrasonography showing a homogeneous, low-echoic mass measuring 24 mm, originating from the fourth layer of the gastric wall (B). Follow-up EGD at four months of follow-up showing no significant changes in the size or morphology of the lesion (C). EGD at 12 months of follow-up continued to show no significant changes (D). EGD at 24 months of follow-up showing a depressed area in part of the tumor, prompting further evaluation (E). EGD showing a bleeding ulcerated tumor (F).

**Figure 5:**
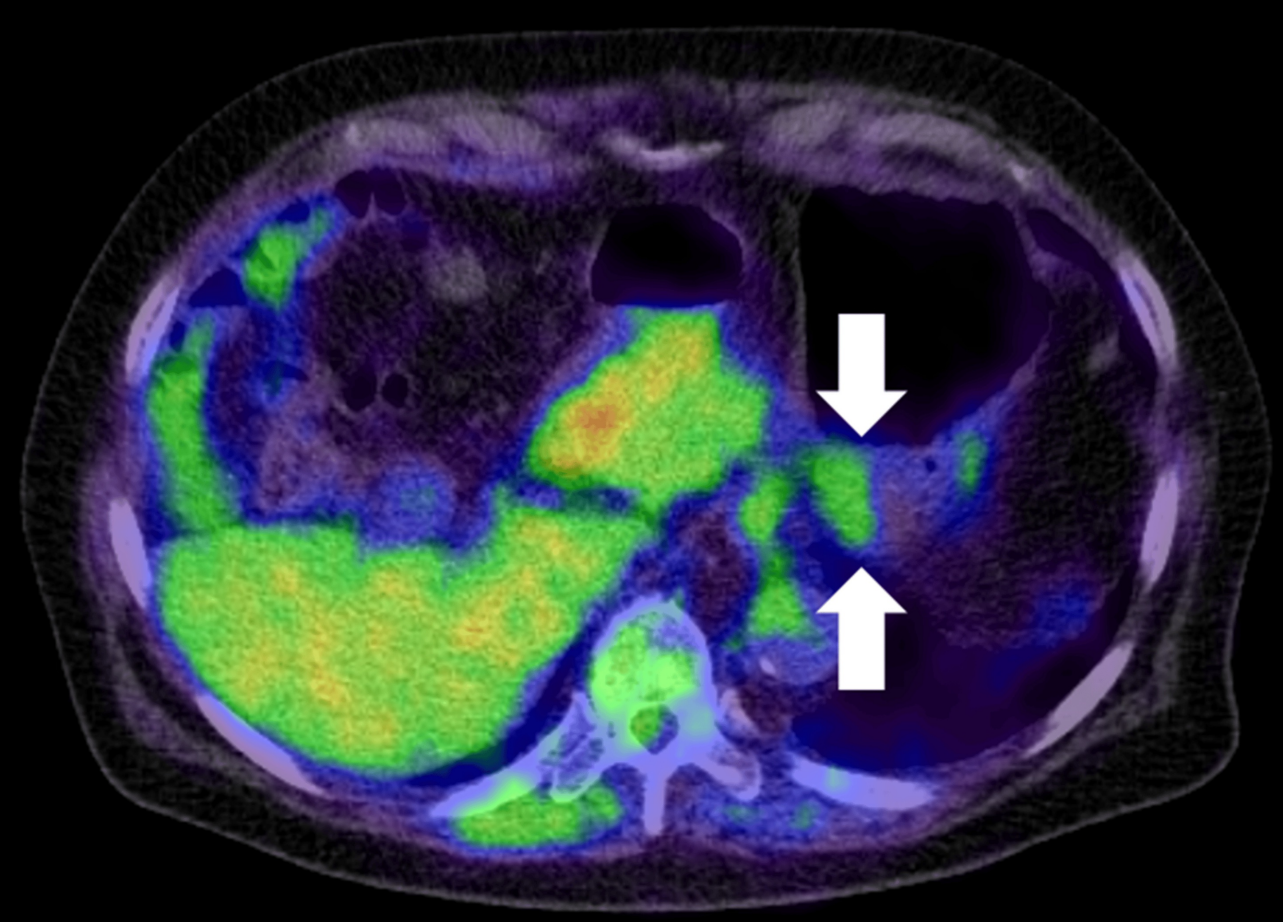
Positron emission tomography (PET) of case 2. The PET showing a maximum standardized uptake value (SUVmax) of 2.92 in the gastric lesion (arrows), which is suggestive of a benign lesion rather than a gastrointestinal stromal tumor.

Esophagogastroduodenoscopy performed at our institution revealed a 30 mm SEL in the gastric fundus with firm elasticity upon palpation with a cannula (Figure 4A). Endoscopic ultrasonography revealed a homogeneous, low-echoic mass measuring 24 mm, originating from the fourth layer of the gastric wall (Figure 4B). Positron emission tomography revealed a maximum standardized uptake value of 2.92 (Figure 5, arrows). Owing to the relatively low tracer uptake and absence of endoscopic ultrasonography features indicative of GIST, the tumor was suspected to be a leiomyoma rather than a GIST. Therefore, the SEL was managed with follow-up observations. Esophagogastroduodenoscopy at four (Figure 4C) and 12 (Figure 4D) months revealed no changes in tumor size or morphology. However, at 24 months, esophagogastroduodenoscopy revealed the development of a depressed area in part of the tumor (Figure 4E). Given this morphological change, an endoscopic ultrasound-guided fine-needle aspiration biopsy was planned for histological diagnosis. Nevertheless, the patient developed a cerebral infarction the day before the procedure and was hospitalized for treatment in the neurology department of our hospital. Thus, an endoscopic ultrasound-guided fine-needle aspiration biopsy was not performed. One month after discharge, the patient developed another cerebral infarction. Additionally, paroxysmal atrial fibrillation was noted, which led to the initiation of rivaroxaban therapy.

**Figure 6:**
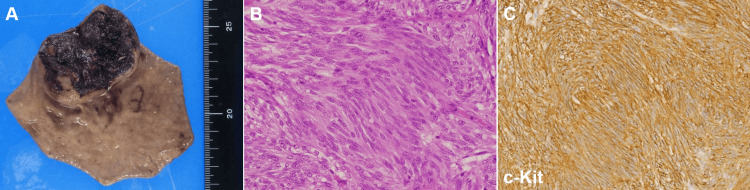
Surgical and histopathological findings of case 2. Laparoscopic partial gastrectomy specimen showing a 27-mm tumor with extensive necrosis and hemorrhage (A). Histological examination showing fascicular proliferation of spindle-shaped cells, consistent with a gastrointestinal stromal tumor (GIST) diagnosis (B). Immunohistochemical staining for c-Kit confirming a diagnosis of GIST (C). The mitotic index was 2 per 50 high-power fields.

Two months later, he developed severe anemia and hematemesis and was urgently transported to our hospital. Esophagogastroduodenoscopy revealed a bleeding ulcerated tumor (Figure 4F), and endoscopic hemostasis was attempted with clips but was unsuccessful, requiring interventional radiology for hemostasis. Subsequent laparoscopic-endoscopic cooperative surgery was performed, and a tumor measuring 27 mm in diameter with extensive necrosis and hemorrhage was resected (Figure 6A). Pathological examination revealed fascicular proliferation of spindle-shaped cells (Figure 6B). The cells were positive for c-Kit (Figure 6C), which was consistent with a diagnosis of GIST. The mitotic index was 2 per 50 high-power fields.

## Discussion

The peak incidence of GISTs occurs between the ages of 60 and 74 years, and it is common for GISTs to be found in older adults [2]. Although surgical resection is generally recommended as the standard treatment for GISTs, older adults often have multiple comorbidities that make surgery less feasible [3,4]. In such cases, surgery may not be performed routinely as in younger patients, and treatment decisions must be carefully considered. One study reported that older adults were significantly less likely to undergo surgery than younger patients, and the rate of adjuvant treatment with imatinib was lower among the older adult population [3]. Additionally, older adults are more likely to discontinue imatinib because of drug-related toxicity, which can negatively affect their prognosis. Furthermore, older adults have a higher rate of postoperative complications and are less likely to receive adjuvant therapy, highlighting the challenges in treatment selection for this age group [4].

In the two cases presented, both patients were initially scheduled for surgical resection or biopsy, but the planned treatments were canceled owing to the onset of cerebral infarction in each patient. Therefore, a conservative observational approach was adopted. Subsequently, both tumors progressed, leading to hemorrhage, and the patient underwent resection. Although previous studies have suggested that some small GISTs remain stable over time [5-8], our cases demonstrate that tumor growth can occur, leading to ulceration and bleeding. The initial tumor size of gastric SELs is a key risk factor for growth, with larger SELs being more likely to increase in size. Studies suggest that tumors >9.5-20 mm have a higher risk of progression [7-11], although the thresholds vary, highlighting the need for tailored surveillance based on tumor size. In both cases, the initial tumor size exceeded these thresholds and endoscopic follow-up confirmed tumor enlargement over time.

The reported growth rate of gastric SELs varies widely, ranging between 2.0-28.4%, depending on study design [5-10,12]. Larger studies encompassing all gastric SELs have reported relatively lower growth rates of 2.0-8.5% [5-8,12], whereas smaller studies focusing specifically on histologically confirmed gastric GISTs or lesions suspected of being GISTs on endoscopic ultrasonography have demonstrated broader growth rate ranges of 5.4-28.4% [6,13]. The doubling time of GISTs has been reported as 17.2 or 19.2 months, with higher-risk tumors doubling in less than six months [14-17]. Regardless of the specific growth rate, it is important to note that enlarging GISTs, as observed in our cases, can lead to tumor-related hemorrhage. In both cases, the patients received antithrombotic therapy for secondary prevention of cerebral infarction recurrence, which may have further increased the risk of bleeding. Indeed, GIST evaluation and treatment have been delayed owing to stroke management, a scenario that commonly occurs in clinical practice. These cases illustrate the potential risks of tumor progression and hemorrhage in patients with gastric GISTs under conservative management.

In case 1, endoscopic ultrasonography revealed a tumor with an irregular border and a heterogeneous internal echo pattern, features known to be associated with an increased risk of GIST growth [17-19]. In contrast, case 2 initially presented as a homogeneous, low-echoic mass with smooth margins, suggestive of a benign lesion such as a leiomyoma. However, during follow-up, the tumor developed a depressed area, indicating morphological changes that warrant further evaluation. This case highlights the importance of serial endoscopic monitoring, as tumors with initially benign characteristics on endoscopic ultrasonography may undergo significant morphological changes. Although guidelines for endoscopic surveillance assume asymptomatic gastric GISTs smaller than 2 cm, the National Comprehensive Cancer Network [20] and Japanese guidelines [21] recommend examinations every six to 12 months, while the European Society for Medical Oncology guidelines [22] suggest an initial short-term follow-up, such as three months, with extended intervals if no growth is observed. These recommendations provide a useful reference for determining appropriate endoscopic surveillance intervals.

It is noteworthy that in case 1, although no mitotic figures were observed in the initial histopathological evaluation, the resected specimen showed 16 mitotic figures per 50 high-power fields. The Ki-67 labeling index increased from 1.9% to 9.7%. Since the initial pathological diagnosis was based on a small fine-needle aspiration specimen, the possibility of sampling bias cannot be ruled out. However, this finding suggests that the mitotic count of GIST may increase over time. Given that mitotic count is a crucial prognostic factor, with higher counts associated with increased mortality risk [23], earlier resection when the mitotic count is still low may contribute to improved prognosis in GIST.

## Conclusions

Gastric GISTs can progress over time, increasing the risk of ulceration and bleeding, particularly in patients receiving anticoagulant therapy. Growth rates vary widely depending on study design and patient selection, with high-risk tumors exhibiting a greater likelihood of enlargement. For older adults with significant comorbidities who are under conservative observation, close follow-up is essential, and early intervention should be considered in cases of tumor enlargement or morphological changes. A multidisciplinary approach is required to ensure optimal management and prevent avoidable complications in high-risk patients with GISTs.

## References

[1] Deprez PH, Moons LM, OʼToole D (2022). Endoscopic management of subepithelial lesions including neuroendocrine neoplasms: European Society of Gastrointestinal Endoscopy (ESGE) Guideline. Endoscopy.

[2] Khan J, Ullah A, Waheed A (2022). Gastrointestinal stromal tumors (GIST): a population-based study using the SEER database, including management and recent advances in targeted therapy. Cancers (Basel).

[3] Farag S, van Coevorden F, Sneekes E (2017). Elderly patients with gastrointestinal stromal tumour (GIST) receive less treatment irrespective of performance score or comorbidity - a retrospective multicentre study in a large cohort of GIST patients. Eur J Cancer.

[4] Yang Z, Feng X, Zhang P (2019). Clinicopathological outcomes and prognosis of elderly patients (≥ 65 years) with gastric gastrointestinal stromal tumors (GISTs) undergoing curative-intent resection: a multicenter data review. J Gastrointest Surg.

[5] Iwamuro M, Okada H, Otsuka M (2025). Natural course and long-term outcomes of gastric subepithelial lesions: a systematic review. J Clin Med.

[6] Lim YJ, Son HJ, Lee JS (2010). Clinical course of subepithelial lesions detected on upper gastrointestinal endoscopy. World J Gastroenterol.

[7] Song JH, Kim SG, Chung SJ, Kang HY, Yang SY, Kim YS (2015). Risk of progression for incidental small subepithelial tumors in the upper gastrointestinal tract. Endoscopy.

[8] Ye LS, Li Y, Liu W, Yao MH, Khan N, Hu B (2020). Clinical course of suspected small gastrointestinal stromal tumors in the stomach. World J Gastrointest Surg.

[9] Fang YJ, Cheng TY, Sun MS, Yang CS, Chen JH, Liao WC, Wang HP (2012). Suggested cutoff tumor size for management of small EUS-suspected gastric gastrointestinal stromal tumors. J Formos Med Assoc.

[10] Kobayashi R, Hirasawa K, Ozeki Y (2024). Clinical course of small gastric subepithelial lesion less than 20 mm diagnosed by endoscopic ultrasound-guided fine-needle aspiration. J Gastroenterol Hepatol.

[11] Hernandez-Lara AH, de Paredes AG, Song LM (2021). Outcomes of endoscopic ultrasound and endoscopic resection of gastrointestinal subepithelial lesions: a single-center retrospective cohort study. Ann Gastroenterol.

[12] Iwamuro M, Mitsuhashi T, Inaba T (2024). Results of the interim analysis of a prospective, multicenter, observational study of small subepithelial lesions in the stomach. Dig Endosc.

[13] Hu ML, Wu KL, Changchien CS, Chuah SK, Chiu YC (2017). Endosonographic surveillance of 1-3 cm gastric submucosal tumors originating from muscularis propria. World J Gastroenterol.

[14] Koizumi S, Kida M, Yamauchi H (2016). Clinical implications of doubling time of gastrointestinal submucosal tumors. World J Gastroenterol.

[15] Ueyama T, Kawamoto K, Iwashita I (1995). Correlation between tumor volume doubling time and histologic findings in gastric smooth muscle tumors: clinical implications of tumor volume doubling time. J Surg Oncol.

[16] Hata S, Arai M, Suzuki T (2013). Clinical significance of endoscopic ultrasound for gastric submucosal tumors. Clin Res Hepatol Gastroenterol.

[17] Shiratori W, Matsumura T, Okimoto K (2023). Long-term course of gastric submucosal tumors: growth speed and size-increasing factors. Gastrointest Endosc.

[18] Melzer E, Fidder H (2000). The natural course of upper gastrointestinal submucosal tumors: an endoscopic ultrasound survey. Isr Med Assoc J.

[19] Lok KH, Lai L, Yiu HL, Szeto ML, Leung SK (2009). Endosonographic surveillance of small gastrointestinal tumors originating from muscularis propria. J Gastrointestin Liver Dis.

[20] von Mehren M, Kane JM, Riedel RF (2022). NCCN Guidelines® Insights: gastrointestinal stromal tumors, version 2.2022. J Natl Compr Canc Netw.

[21] Hirota S, Tateishi U, Nakamoto Y (2024). English version of Japanese Clinical Practice Guidelines 2022 for gastrointestinal stromal tumor (GIST) issued by the Japan Society of Clinical Oncology. Int J Clin Oncol.

[22] Casali PG, Blay JY, Abecassis N (2022). Gastrointestinal stromal tumours: ESMO-EURACAN-GENTURIS Clinical Practice Guidelines for diagnosis, treatment and follow-up. Ann Oncol.

[23] Steigen SE, Eide TJ (2006). Trends in incidence and survival of mesenchymal neoplasm of the digestive tract within a defined population of northern Norway. APMIS.

